# Predicting ^31^P NMR Shifts in Large-Scale, Heterogeneous Databases by Gas Phase DFT: Impact of Conformer and Solvent Effects

**DOI:** 10.1021/acsomega.5c13249

**Published:** 2026-01-16

**Authors:** Robert Geitner, Christian Dreßler

**Affiliations:** † Group for Physical Chemistry/Catalysis, Department of Mathematics and Natural Sciences, Institute of Chemistry and Bioengineering, Technische Universität Ilmenau, Weimarer Str. 32, 98693 Ilmenau, Germany; ‡ Group for Solid State Physics, Department of Mathematics and Natural Sciences, Institute of Physics, Technische Universität Ilmenau, Weimarer Str. 32, 98693 Ilmenau, Germany

## Abstract

In this study, we extend the Ilm-NMR-P31 data set by systematically enriching experimental ^31^P nuclear magnetic resonance (NMR) data with quantum-chemically derived information. Using density functional theory (DFT) at the BLYP and B3LYP levels, we calculated ^31^P chemical shifts and optimized molecular geometries for 10,007 phosphorus-containing compounds. This hybrid data set provides a consistent framework that combines experimental measurements with theoretical descriptors, thereby facilitating the development of improved machine learning (ML) models and advanced structure elucidation tools. To evaluate the reliability of the computational data, we compared the DFT-derived shifts against experimental values. Single-point calculations in vacuum resulted in a root mean squared error (RMSE) of 30.82 ppm, which improved to 29.37 ppm when up to 20 conformers per molecule were included. Implicit solvent models produced minor additional improvements. These findings highlight the importance of conformational sampling. Evaluation of different functionals on the Ilm-NMR-P31 database, including those closely reproducing coupled-cluster results, demonstrated that B3LYP not only achieves sufficient accuracy for reproducing NMR shifts across large experimental databases but also emerges as a broadly applicable choice for routine applications. Overall, this work provides a large-scale, quantum chemically enriched ^31^P NMR data set that enhances the value of experimental data and supports future ML-based prediction models.

## Graphical abstract

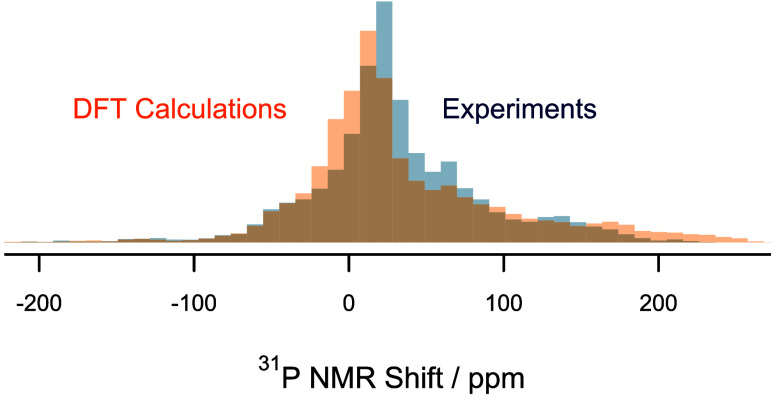

## Introduction

Machine learning techniques are currently being widely investigated for applications in chemistry.^1–4^ One of the first areas where early machine learning algorithms were used was in computer-assisted structure elucidation (CASE) systems.,^5,6^ e.g. Nuzillard and Georges presented their “Logic for structure determination” in 1991 to automatically derive structures from a given set of NMR spectra.^7^ One important step in CASE systems is the prediction of NMR spectra to compare predicted spectra of a potential candidate molecule to the experimental spectra. Nowadays, graph neural networks (GNNs) are the leading approach when it comes to NMR shift prediction via machine learning algorithms.^8^

For ^1^H and ^13^C NMR spectroscopy, large and well-curated data sets are readily available, such as nmrshiftdb2.^9^ In contrast, data availability for heteronuclei is much more limited. To address this gap, we recently introduced the Ilm-P31-NMR data set, which currently contains 13,730 unique phosphorus-containing compounds with experimental ^31^P NMR chemical shifts and partly with associated metadata such as solvent and measurement temperature.^10^ While this data set represents a valuable starting point, we recognized that further annotation is necessary to train more robust and generalizable machine learning models.

A promising alternative is to enrich experimental data with complementary information obtained from quantum chemical calculations. Density functional theory (DFT) has proven to be a reliable method to compute NMR shielding constants via the Ramsey formalism.^11,12^ Unlike purely data-driven approaches, DFT provides a bottom-up description of molecular electron densities and their influence on NMR observables.

DFT calculations were used in many studies to predict the relevant NMR shifts. For example, Gao et al. studied different classes of phosphorus containing molecules using DFT/Gauge-Independent Atomic Orbitals (GIAO) methods and achieved a root-mean-square error (RMSE) of 5.32 ppm for 35 molecules compared to experimental values.^13^ Payard et al. studied 25 transition metal complexes and their phosphine ligands on the B3LYP level of theory and achieved a RMSE of 12.0 ppm compared to experimental values.^14^ In another example Kondrashova et al. used PBE0 as functional in conjunction with different basis sets to describe ^31^P NMR shifts in 39 Pd complexes achieving a RMSE of 6.9 ppm compared to experimental values.^15^

It is well-known that the NMR shielding value is strongly dependent on the molecule’s geometry. Thus, modern structure elucidation algorithms do not relay on a single conformer, but on a conformer ensemble from which Boltzmann-weighted NMR shifts can be calculated.^16^ Besides the influence of conformers, the choice of functionals and basis sets is also important.^17,18^ Maryasin and Zipse as well as Schattenberg and Kaupp studied the influence of functionals on ^31^P shifts,^19,20^ while Shenderovich investigated the influence of the basis set on the accuracy of ^31^P NMR shift calculations.^17^ The final important factor for NMR shift calculations is the choice of a solvent model. Maryasin and Zipse studied the effect of solvents on ^31^P NMR shifts using explicit solvent models,^19^ a work on which Calcagno et al. based their molecular dynamics simulations.^21^ They found a maximum shift range of 14.0 ppm for OPPh_3_. Both studies identified that explicit solvent models are better than simple implicit solvent models, although they come at a much greater computational cost. Streck and Barnes studied the influence of different solvents on the ^31^P NMR signal of trimethyl phosphate experimentally.^22^ They found that the ^31^P shift varies between 1.9 and 4.3 ppm with an average of 3.5 ppm for 24 solvents.

As can be seen from the discussed examples, studies usually focus on small, highly specific groups of molecules. While these results are interesting for a specific application they do not generalize well to a large data set. We could to the best of our ability not find any report on the large-scale calculation of ^31^P NMR properties. The closest data set we found is the *kraken* library from Gensch et al. how used DFT on 1558 phosphines and their Ni complexes to build a comprehensive phosphine database.^23^

As a consequence of the size and heterogeneity of the Ilm-NMR-P31 data set, the experimental conditions and protocols vary considerably between compounds. Among these factors, the solvent is by far the most heterogeneous and also one of the most influential on the observed ^31^P NMR shifts. Although four different solvents are explicitly reported in the database, for the majority of entries the solvent is not specified.

It is important to emphasize that solvent effects on ^31^P chemical shifts are typically on the order of several ppm^22^—the same order of magnitude as the differences observed between commonly used density functionals.^19,20^ Consequently, uncertainty about the solvent often dominates over functional choice in determining the absolute accuracy of computed shifts.

The first goal of this article is therefore to evaluate to what extent gas-phase DFT calculations can reproduce measured NMR shifts across a large, heterogeneous data set. We will examine which computational settings are best suited for such comparisons, and whether including implicit solvent models or conformer ensembles — to capture geometric variability — can improve agreement with experiments.

At present, machine-learning prediction of ^31^P NMR shifts is mainly established for solids. The ShiftML family^24,25^ achieves DFT-level accuracy for chemical shifts in molecular solids and is widely used. In contrast, prediction of ^31^P NMR spectra in liquids is still less mature; here, DFT calculations with careful referencing and approximate solvent treatment remain the practical baseline.

The Ilm-NMR-P31 data set was explicitly designed to enable ML on liquid-state ^31^P data. In its present form, the data set primarily allows mapping between SMILES strings and experimental ^31^P chemical shifts. State-of-the-art ML methods for atomistic properties — such as equivariant GNNs or Gaussian process regression — rely on local structural descriptors (e.g., Atomic Cluster Expansion (ACE) or Smooth Overlap of Atomic Positions (SOAP)), which are also the foundation of ShiftML for solid-state NMR.

It is therefore highly likely that future ML approaches for liquid-phase ^31^P NMR will also require local atomic descriptors and training data that explicitly link 3D conformations to observed chemical shifts. At present, such structure–property data is not available for a large number of phosphorus-containing compounds.

The second main goal of this work is thus to close this gap by providing, for more than 10,000 P-containing molecules, multiple conformers per compound together with DFT-calculated ^31^P NMR shifts. This enriched data set will enable the development and training of next-generation ML models for liquid-state ^31^P NMR shift prediction.

The aim of this study is neither to propose a novel computational workflow nor to perform a systematic benchmark of different functionals and basis sets for ^31^P NMR prediction. Rather, the goal is to assess whether NMR shifts recorded in solution from large, heterogeneous databases — often with incomplete metadata such as unknown solvents — can still be reasonably reproduced by gas-phase DFT calculations, and to evaluate whether the incorporation of implicit solvent models or conformer ensembles to capture geometric variability can improve the agreement in this level of theory–experiment comparison.

In addition, we aim to provide training data from a large-scale data set that links 3D conformations to observed ^31^P chemical shifts, thereby enabling the development of next-generation machine-learning models for liquid-state ^31^P NMR prediction.

## Results and Discussion

NMR shifts for 10,007 molecules from the Ilm-NMR-P31 data set were successfully calculated. This includes geometry optimization at the BLYP level as well as NMR shielding constant calculations at the B3LYP level of theory (for details see the Computational Details section). Both levels were chosen to strike a compromise between accuracy and calculation time. On average a geometry optimization in vacuum took 16.6 min using 16 CPUs (Intel Xeon Platinum 8462Y, 2.8 GHz) starting from the rdkit^26^ generated xyz structures. The subsequent single point calculation took 2.0 min. In total this means that a DFT level NMR spectrum can be obtained in 18.6 min when starting from a SMILES string. This is fine when considering tenth of molecules for a structure elucidation but is not yet on the level that DFT calculation can be used to routinely scan large data sets with thousands or even tens of thousands of molecules. When considering implicit solvent models, we found that the calculation time is increasing between 14% (toluene) and 33% (water) for the geometry optimizations and by 4–7% for the single point NMR calculations when compared to the calculations in vacuum (see Tables 1 and S1 provides an extended view based on the system size).

**Table 1:** Influence of Implicit Solvent Models on the DFT-Derived Prediction Accuracy of ^31^P NMR Shifts^c^

exp. solvent	#of mol.	impl. solvent	RMSE/ppm	MAE/ppm	impr.	calc. time^a^/min
-	10,007	-	30.82	18.22	4.7%	18.6
		Boltzm.-weight.	**29.37**	16.80		498^b^
CHCl_3_	2955	-	26.92	15.42	1.7%	22.6
		CHCl_3_	**26.47**	13.90		
DMSO	43	-	26.46	20.04	4.8%	23.3
		DMSO	**25.19**	12.81		
Toluene	11	-	25.33	23.94	9.1%	21.1
		toluene	**23.03**	21.75		
H_2_O	205	-	30.33	15.09	6.0%	24.4
		H_2_O	**28.50**	14.08		
CH_3_CN	4	-	**39.86**	25.89	–2.3%	24.1
		CH_3_CN	40.79	25.53		

a Geometry optimization + NMR single point calculation, averaged across all 10,007 molecules.

b + Conformer analysis.

c Bold values highlight the best agreement with the mean experimental value. The RMSE values are also used to calculate the relative improvement (impr.).

In addition to the time needed for geometry optimization and single point NMR calculation, the time for a conformer generation and evaluation must be considered. As found by Ermanis et al. at least 20 conformers must be evaluated to capture the influence of thermally induced structure changes like rotations well.^27^ In our data set which includes molecules with up to 80 atoms, conformer generation on average took 32.5 min to generate 355 conformers, which are accessible within a 6 kcal mol^–1^ window at 298.15 K. We only considered up to 20 conformers for a series of geometry optimizations followed by single point calculations to assign a NMR shift to each conformer structure. As 15 conformers were considered on average per molecule, the geometry optimization and follow-up NMR shift calculation took 449 min. Thus, the important consideration of conformers adds 481.5 min in total to the calculation time. From SMILES string to a BLYP/B3LYP level Boltzmann-weighted NMR shift 498 min of calculation time are needed. This number underlines that machine learning approaches that deliver a Boltzmann-weighted NMR shift from a SMILES string have the potential to lower the burden when screening large data sets. Machine learning approaches like GNNs have a high upfront training time but make up for it by being very efficient when considering many individual molecules.

Inspired by the extended method comparison of Schattenberg and Kaupp^20^ we compared the functionals KT3, r^2^SCAN, and B3LYP for the ^31^P NMR shift prediction next. Table 2 reveals a small improvement in relative MAE of 2.5% and 2.2% for KT3 and r^2^SCAN respectively compared to the 2.6% for B3LYP, when comparing the quantumchemical derived ^31^P shifts with experimental values. This improvement is much smaller than the values found by Schattenberg and Kaupp, who found that KT3 and r^2^SCAN are much better than B3LYP. Our explanation for this finding is that Schattenberg and Kaupp compared the functionals against coupled cluster calculations while we chose experimental ^31^P values as reference. The Ilm-NMR-P31 data set is very heterogeneous in terms of molecular structures but also in meta data quality, as e.g., the solvent is not always known.

**Table 2:** Method Comparison for the Calculation of 10.007 ^31^P NMR Shifts^a^

Metric	B3LYP	KT3	r^2^SCAN
RMSE/ppm	30.82	28.35	27.28
MAE/ppm	18.22	17.08	15.27
MSE/ppm	–3.30	2.14	–1.65
rel. MAE/%	2.6	2.5	2.2

a The quantum chemical derived values are compared to experimental values to get the four error measures.

As the performance of B3LYP, KT3, and r^2^SCAN is comparable for the present benchmark, we selected B3LYP as the reference functional. This choice prioritizes methodological transferability and compatibility with existing quantum-chemical data over marginal gains in accuracy. B3LYP is routinely used in electronic-structure studies where NMR parameters are computed as auxiliary observables, enabling straightforward comparison and extension of the data set without method-specific recalculations. Large external resources reporting heteronuclear NMR shifts alongside other molecular properties^23^ illustrate the practical relevance of this approach, even when different computational protocols are employed.

Our improved Ilm-NMR-P31 data set enables us to compare experimentally recorded ^31^P NMR shifts with DFT-level data on multiple levels. Figure 1a shows a pairs plot for the DFT-level data in vacuum against the experimental data. As can be seen the agreement is very good resulting in a RMSE of 30.82 ppm. This value is larger than the literature reported values of Gao et al. (5.32 ppm, 35 molecules)^13^ and Payard et al. (12.0 ppm, 25 transition metal complexes).^14^ We explain this finding with the much larger size and molecular variation of our data set (10,007 molecules) compared to the highly specialized studies. While a finetuning to a specific assignment problem is well advised, a general prediction like in the present case cannot a priori consider each specialty.

**Figure 1:**
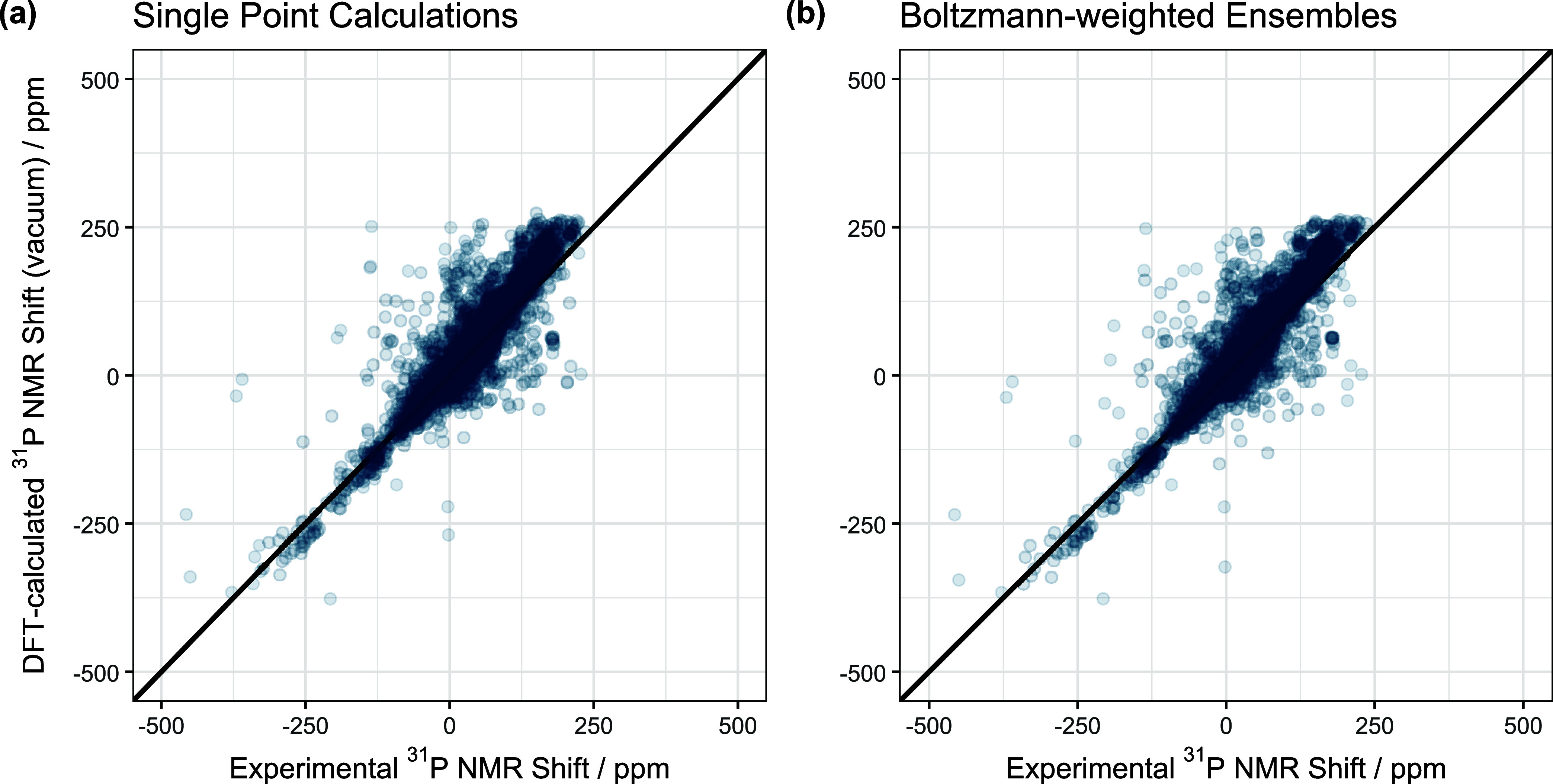
Pairs plot for the comparison of single point derived NMR shifts (a) and Boltzmann-weighted ensemble-based NMR shifts (b) vs experimentally recorded NMR shifts.

As can be seen from Figure 1 some molecules are difficult to describe correctly via the chosen DFT approach. Figure 2 showcases four selected structures and their NMR shifts. It is difficult to pinpoint the molecular origin for the large deviations between experimental and DFT-calculated NMR shift. All structures in Figure 2 have in common that the P atom is surrounded by electronegative atoms like oxygen or nitrogen. In the first three cases the DFT calculation overestimates the deshielding effect, while for the third structure the deshielding is underestimated. In the fourth case one could argue that selenium is an uncommon element and that this might be the origin for the large deviation. Our study does not give a conclusive result on an individual molecule basis but indicates that in-depth DFT or even molecular dynamics simulation are needed to better describe ^31^P NMR shifts for specific molecular structures.

**Figure 2:**
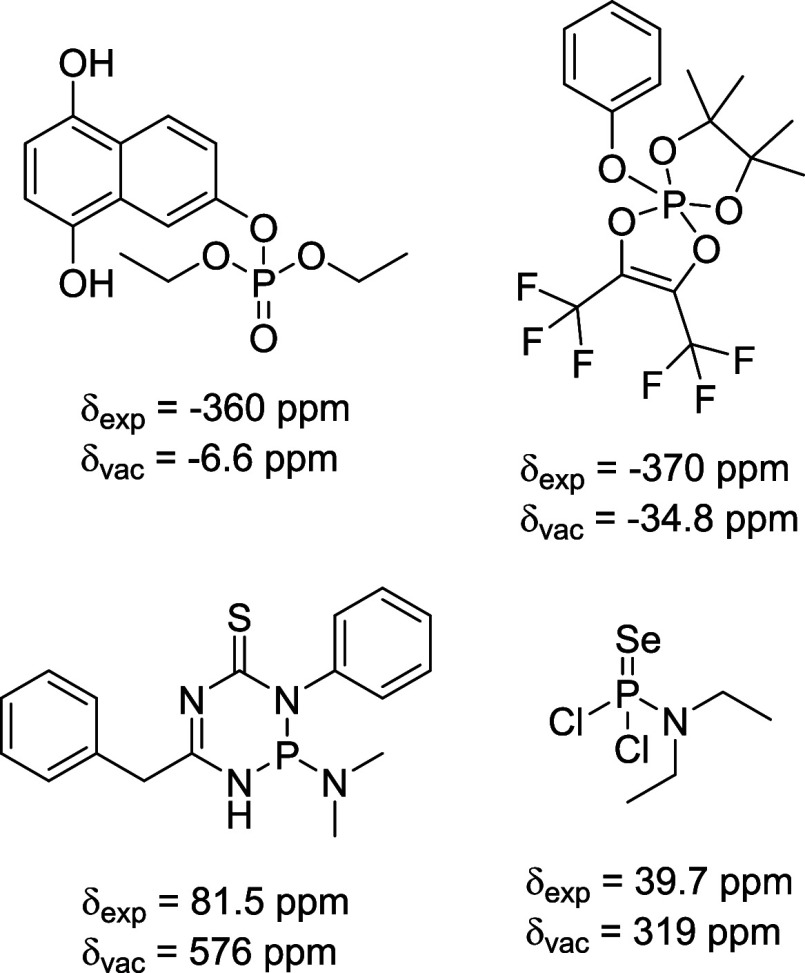
Selected molecules for which the DFT-calculated NMR shift δ_*vac*_ does not match the experimental ^31^P shift δ_exp_ well.

Next, we wondered if a conformer ensemble-based prediction is worth the additional calculation time. Figure 1b and Table 1 summarize our findings. As can be seen the conformer ensemble-based prediction lowers the RMSE to 29.37 ppm, indicating that at least for the ^31^P NMR shifts considered here a conformer ensemble-based prediction is an improvement. When splitting the data set into five quantiles depending on the number of atoms in the molecules, it becomes clear that the largest molecules profit the most from this prediction method (relative improvement of RMSE of 11.6%). Table 3 summarizes this finding for all quantiles while Table S2 provides a similar analysis but uses the number of rotatable bonds to build the five quartiles.

**Table 3:** Correlation between Molecule Size and DFT-Derived Prediction Accuracy of ^31^P NMR Shifts

quantile	size	atom range	RMSE_bol_/ppm	RMSE_vac_/ppm	μ(# of conf.)	μ(rotatable bonds)	calc. time/min
Q1	2002	3–25	37.2	38.5	24	1.77	5.2
Q2	2002	25–32	29.5	30.7	103	3.25	9.8
Q3	2001	32–40	27.3	28.5	244	4.35	15.1
Q4	2001	40–48	27.2	27.9	472	5.32	23.3
Q5	2001	48–80	23.9	27.1	933	6.71	39.7

Our results indicate that conformer consideration for ^31^P NMR shift prediction on the DFT level becomes more important when the molecules of interest are large and flexible. This result aligns with general perception that more degrees of freedom lead to larger geometric deviations between different conformers and thus also between the ^31^P shifts. We conclude that conformer analysis is most important for larger molecules consisting of 48+ atoms.

Our data sets also enable an evaluation of implicit solvent models on DFT-calculated ^31^P NMR shifts. Implicit solvation models play an important role in quantum chemistry. Without the need to explicitly include multiple solvation shells of solvent molecules, these models can effectively mimic the influence of a specific solvent on the solute. Implicit solvent models have the advantage that they are computationally cheap and thus do not extend the calculation time much. Table S1 quantifies this increase to 6–34% additional calculation time depending on the specific solvent and the size of the molecule. The disadvantage is that implicit solvent models are only a simplistic abstraction of the real molecular behavior where solvent molecules might interact with probe molecules via e.g., hydrogen bonding beyond a simple dielectric field. In our case, where more than 10,000 molecules are considered, implicit models are the only valid option to study the influence of a solvent on the ^31^P NMR shift.

Tables 1 and S3 and Figure 3 summarize our results. As can be seen the addition of an implicit solvent model leads to a better prediction of the ^31^P shift, e.g. the RMSE drops from 26.92 to 26.42 ppm when considering chloroform as solvent, which is an improvement by 1.7%. We saw the largest improvement for toluene (9.1%) while for acetonitrile we even saw a slight decline in prediction accuracy of −2.3%, although this effect can most likely be attributed to the small sample size. Overall, the addition of an implicit solvent model improves the DFT-derived ^31^P NMR shift and thus should be a standard procedure when calculating ^31^P shifts, especially as the computational cost is low. The relative improvement in prediction accuracy is larger for the conformer-based prediction (4.7%) than for the implicit solvent model (2.0%), but it comes at the cost of a much-increased calculation time.

**Figure 3:**
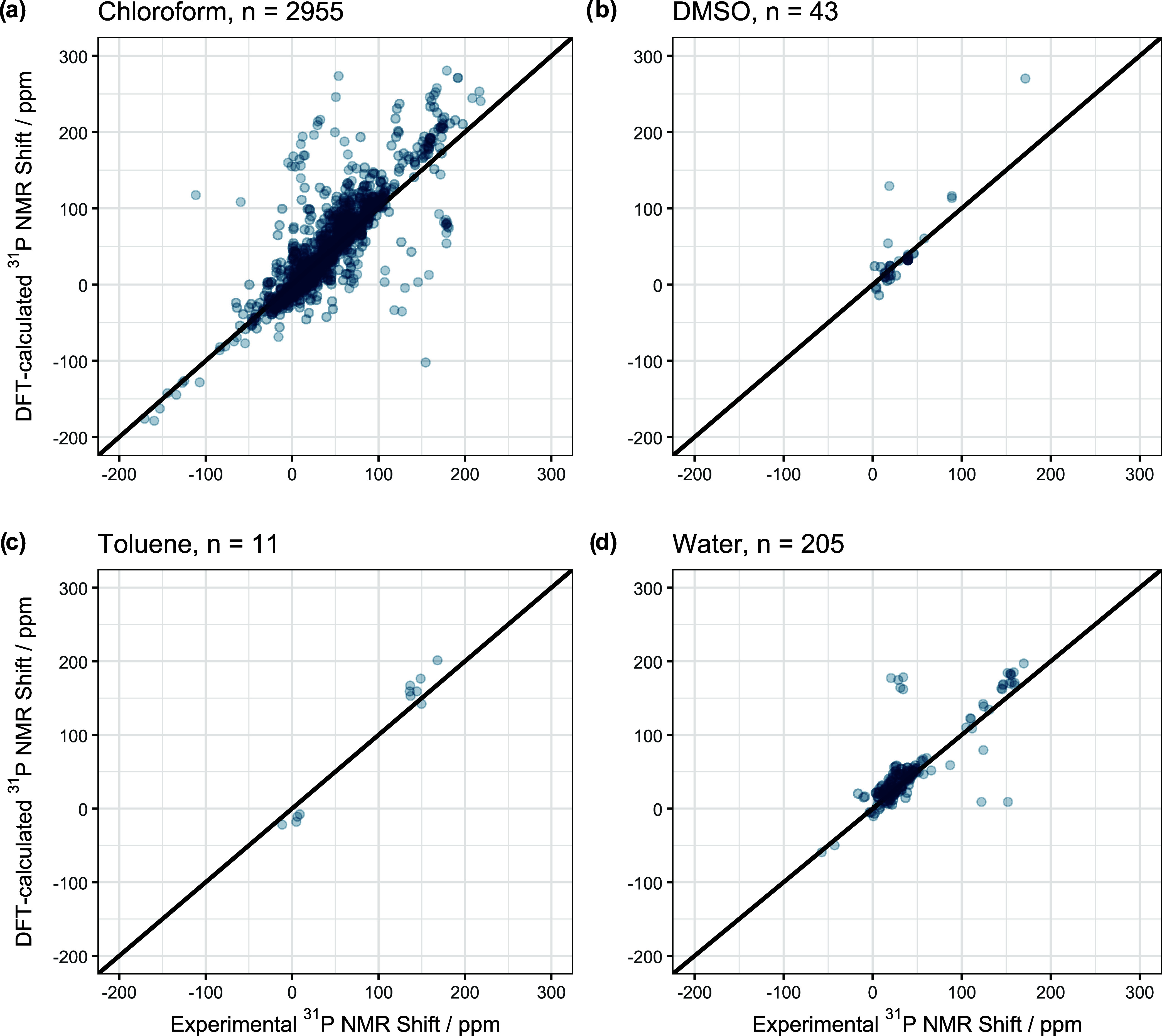
Pairs plot for the comparison of single point derived NMR shifts using different implicit solvent models: (a) Chloroform, (b) DMSO, (c) toluene and (d) water. *n* describes the number of molecules in each cohort.

Furthermore, the increase in prediction accuracy does not correlate with the relative dielectric constant ε_r_ of the solvents. In our study water and toluene showed the largest increase and they are either very polar (80.4) or nonpolar (2.4). For toluene this improvement is most likely linked to the small sample size of only 11 molecules. When leaving out toluene and acetonitrile due to the small sample size, we see that polar solvents like water and DMSO profit more from the CPCM implicit solvent model than the nonpolar chloroform. This result was somewhat surprising to us because although electrostatic effects make up a larger proportion of the influence of polar solvents we expected that leaving out explicit interactions like hydrogen bonds causes more issues for polar than nonpolar solvents.

When looking at classes of phosphorus containing molecules monoalkyl phosphinates and phosphonites show the largest increase in prediction accuracy (RMSE improvement by 30.7 and 8.7%, respectively), while phosphonates and dialkyl phosphinates even got worse (−3.7% and −26.7%) when an implicit solvent—water in these cases—was considered in the prediction (see Table 4). The DFT-derived values are sometimes close to the experimental values and sometimes are off by a large margin as is the case for phosphonites. Although the implicit solvent model improved the prediction the shift values were still far off the experimental ones. This trend is identical for each solvent we studied: Some molecule classes profit more than others, but there is no clear trend that e.g., polar molecules profit more in polar solvents.

**Table 4:** Influence of an Implicit Water Solvent Model on DFT-Calculated ^*31*^P NMR Shifts by Molecular Class^a^

class	# of mol.	μ(δ_exp_)/ppm	μ(δ_vac_)/ppm	μ(δ_H2O_)/ppm
monoalkyl phosphinates/RCH_2_P(=O)(H)OR	15	24.7	21.4	**23.1**
phosphonites/RCH_2_P(OR)_2_	16	115.2	178.4	**173.4**
phosphonates/RCH_2_P(=O)(OR)_2_	58	18.3	**15.7**	19.3
dialkyl phosphinates/(RCH_2_)_2_P(=O)OR	72	34.3	**34.3**	42.4

a Some molecule classes profit from the addition of an implicit solvent model, while others perform worse when compared to the calculation in vacuum. Bold values highlight the best agreement with the mean experimental value.

## Conclusion

In this study, we present a comprehensive evaluation of DFT-derived ^31^P NMR chemical shifts for 10,007 mainly organophosphorus compounds from the Ilm-NMR-P31 data set. By combining BLYP-level geometry optimizations with B3LYP-based shielding calculations, we achieved a practical balance between computational cost and accuracy, requiring on average 18.6 min per molecule. Incorporating conformer ensembles increases the total runtime to 498 min per molecule, but improves predictive performance, reducing the RMSE from 30.82 to 29.37 ppm. This improvement is particularly pronounced for large molecules, underlining the importance of conformational flexibility in NMR shift prediction.

We further demonstrate that implicit solvent models can enhance prediction accuracy with minimal additional computational burden. While the improvements are modest overall, specific solvents like toluene yielded up to 9.1% RMSE reduction. However, the benefit of solvent correction does not correlate with solvent polarity, suggesting that specific solute–solvent interactions — beyond the scope of implicit models — play a crucial role in certain systems. Furthermore, solvent effects are highly class-dependent, with some compound classes showing substantial improvement, while others worsen.

Despite the methodological advancements, selected individual compounds remain difficult to describe using standard DFT approaches. These outliers indicate the limitations of current models and suggest that explicit solvation or higher-level treatments (e.g., molecular dynamics or hybrid solvation schemes) may be required for accurate prediction in specific cases.

Taken together, our findings highlight both the capabilities and limitations of current DFT methods in large-scale ^31^P NMR prediction. They also underscore the potential of machine learning approaches to accelerate screening and shift prediction, particularly when trained on high-quality DFT-derived data sets such as our Ilm-NMR-P31 data set. Future developments should focus on hybrid strategies that combine quantum chemical accuracy with machine learning efficiency and the inclusion of explicit solvent effects where needed.

### Computational Details

From the 13,730 present in the Ilm-NMR-P31 data set only 10,007 were considered for DFT calculations. We excluded ion pairs with large anions and cations as well as molecules with elements from the sixth period and beyond. The reason being that both classes would need dedicated treatment either because of larger basis sets or relativistic contributions. The optimized structures contain the elements H (number of molecules with at least one H atom *n* = 9921, mean number of H atoms per molecule μ = 18.12931), Li (37, 0.0037), B (20, 0.0022), C (9963, 12.73419), N (5051, 1.00769), O (7540, 2.26811), F (981, 0.46947), Na (59, 0.0071), Si (809, 0.1458), P (10007, 1), S (2400, 0.31638), Cl (1749, 0.30449), K (8, 0.0012), V (1, 1 × 10^–04^), Ge (47, 0.0061), As (7, 9 × 10^–04^), Se (237, 0.02448), Br (335, 0.04377), Sn (39, 0.0051), Te (18, 0.0019), I (48, 0.0052). An extended discussion of the statistics of the data set can be found in the Supporting Information. No dedicated treatment of relativistic effects was applied. The computational workflow is shown in Figure 4 and is oriented on the work done to construct the *kraken* database.^23^

**Figure 4:**
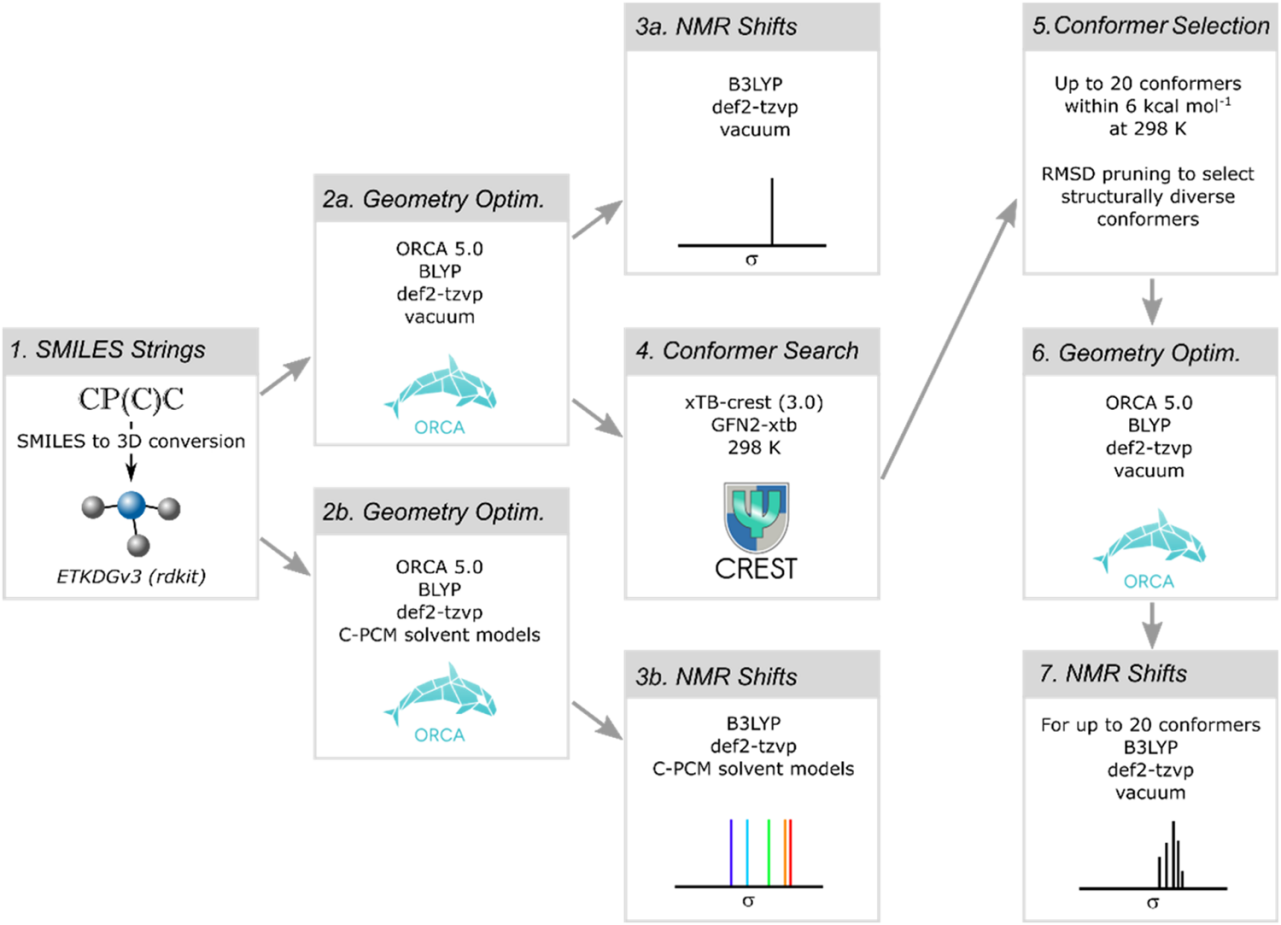
Schematic depiction of the workflow to derive solvent and conformer dependent ^31^P NMR shifts.

To compare experimental (δ_exp_) and DFT-calculated NMR shifts (δ_calc_) we used the root-mean-square error (RMSE) defined as

$$\mathrm{RMSE}=\sqrt{\frac{1}{n}\sum_{i=1}^{n}{\left(\delta_{\exp,i}-\delta_{\mathrm{calc},i}\right)}^{2}}$$

the mean absolute error (MAE) defined as

$$\mathrm{MAE}=\frac{1}{n}\sum_{i=1}^{n}\left|\delta_{\exp,i}-\delta_{\mathrm{calc},i}\right|$$

as well as the mean signed error (MSE) defined as

$$\mathrm{MSE}=\frac{1}{n}\sum_{i=1}^{n}\delta_{\exp,i}-\delta_{\mathrm{calc},i}$$

Initially, the canonical SMILES^28^ strings of 10,007 molecules from the Ilm-P31-NMR data set were converted to xyz files using python and rdkit.^26,29^ The initial structure generation was done using the ETKDGv3 algorithm implemented in rdkit.^30,31^ To verify that the xyz coordinates match the SMILES strings both were used to generate molecular graphs applying rdkit. This verification step showed no mismatches but should be treated cautiously as the same algorithm was also used to generate the xyz coordinates. In addition, a manual inspection of 100 molecules (1%) also verified the structure generation.

The DFT calculations were done using ORCA 5.0.^32–34^ All calculations were performed on the Ilmenau High Performance Computing Cluster using 16 CPU on nodes with Intel Xeon Platinum 8462Y processors running at 2.8 GHz. The generated structures were optimized using the BLYP functional,^35,36^ the def2-TZVP basis set^37^ and employing D4 dispersion correction.^38^ This optimization was done for isolated molecules in the gas phase. For the optimized structures a frequency analysis was conducted to ensure a valid minimum in the PES has been found.^39^ Subsequently, the NMR shielding tensor was calculated for all atoms using the B3LYP functional^36,40^ and the def2-TZVP basis set^37^ applying the Gauge-Independent Atomic Orbital (GIAO) method.^41,42^

Besides B3LYP the KT3 ^43^ and r^2^SCAN^44^ functionals were also tested as possible alternatives based on the method comparison by Schattenberg and Kaupp.^20^ Table 2 shows the results of the method comparison by listing the MAE, the RMSE, the MSE, as well as the relative MAE. The latter is derived by dividing the MAE by the range of ^31^P shifts (Δδ = 698).

To prove that a relativistic treatment yields no significant improvement the NMR shifts were also calculated using the Zero-Order Regular Approximation (ZORA).^45^ This was done for the 105 molecules containing fifth row elements namely Sn, Te and I. For the heavy elements the SARC-ZORA-TZVP basis set^46^ was used while all other elements were described using the ZORA-def2-TZVP^46^ basis set. In addition, the SARC/J auxiliary functions^46^ were also used during relativistic treatment. The D4 dispersion correction^38^ was also applied. The results are shown in Figure S2 and indicate that realistic treatment only leads to marginal improvements.

The calculation process (geometry optimization, frequency analysis, NMR single point calculation) was repeated by adding implicit solvent models in the form of a Conductor-like Polarizable Continuum Model (C-PCM) for chloroform, dimethyl sulfoxide (DMSO), water, toluene and acetonitrile during geometry optimization, frequency calculation as well as during NMR single point calculations. The solvent was simulated by adding a constant electric field with the respective dielectric constant of solvent. It is important to note that the dielectric constants of the nondeuterated solvents were used. As before geometry optimization and frequency analysis was done using the BLYP functional,^35,36^ the def2-TZVP basis set^37^ and employing D4 dispersion correction.^38^ The NMR calculation was done using the B3LYP functional^36,40^ and the def2-TZVP basis set.^37^

For the isolated molecules in the gas phase the DFT optimized structures were used as input to a conformer search using xtb-crest (3.0) and GFN2-xTB.^47,48^ This algorithm searched for all thermo-available states within a 6 kcal mol^–1^ at 298.15 K. For up to 20 conformers the xTB-dervied structures were optimized at the BLYP level of theory before NMR shielding tensors were calculated using again the B3LYP functional and the def2-TZVP basis set in ORCA. In case more than 20 conformers were available in the 6 kcal mol^–1^ range the conformer ensemble was pruned using python code from PyDP4.^49^ A candidate conformer was compared to all already selected conformers by its root mean square deviation of atomic positions (RMSD) values. If the minimal RMSD value was larger than 0.5 Å the candidate conformer was added to the pruned ensemble. This process was repeated until 20 conformers were selected and started from the conformers with the lowest relative energy. For the conformer ensemble the lowest, highest and Boltzmann-averaged NMR shielding values at 298.15 K were calculated

$$\sigma_{\mathrm{bol}}=\frac{\sum_{i}\sigma_{i}\cdot e^{-{\Delta G}_{i}/\mathrm{RT}}}{\sum_{i}e^{-{\Delta G}_{i}/\mathrm{RT}}}$$

σ_*i*_ describes the NMR shielding value of the *i*-th conformer, Δ*G*_*i*_ is the relative Gibbs energy of the *i*-th conformer relative to the Gibbs energy of the conformer with the lowest energy, *R* is the universal gas constant and *T* is the absolute temperature.

To compare the DFT-calculated shielding values σ_*i*_ to the experimental shift values the shielding values were converted to shift values δ_*i*_ by the following equation

$$\delta_{i}=\sigma_{\mathrm{ref}}-\sigma_{i}-\delta_{\mathrm{ref}}$$

As reference the shielding value σ_ref_ of trimethyl phosphine (PMe_3_) was used as the standard 85% phosphoric acid in water is difficult to simulate. To achieve a reference to 85% phosphoric acid in water, all calculated shift values σ_*i*_ were additionally corrected by the experimental shift value δ_*re*f_ of trimethyl phosphine (−62.0 ppm).^50^

Besides the DFT information for 10,007 molecules we also added 66 new single phosphorus containing molecules as well as 1825 components which contain more than one phosphorus atom, but which are symmetric, so that all phosphorus atoms show the same ^31^P NMR shift to the Ilm-NMR-P31 data set. This brings the number of entries in the Ilm-NMR-P31 data set to 15,976.

As described earlier^10^ the data processing was mainly done using R (4.4.3)^51^ and its packages. The MOL files were read into R using the package ChemmineR^52^ and further processed using the package ChemmineOB^53^ and software OpenBabel (3.1.1).^54^ The number of rotatable bonds was calculated using the R package reticulate^55^ and the python package rdkit.^26^ Consequently, the strict rotatable bond definition implemented by rdkit was applied.

The new DFT-derived NMR information (single point values, ensemble shifts, single point values using implicit solvent models) is saved to the data block of the respective SDF file using the tags suggested by the NMReData initiative.^56^ The data is also available as CSV file. The last available format is an R tibble,^57^ a variant of a data frame, which contains the same information as the CSV file as well as the SDF object and the molecular graph object for each molecule.

## Supplementary Material



## Data Availability

The data is available online at the GitHub repository https://github.com/clacor/Ilm-NMR-P31. The data set is also available from Zenodo 10.5281/zenodo.8260783. The relevant graph processing R scripts were published as an independent R package available from GitHub https://github.com/clacor/MolGraphR.

## Notes

Data set statistics; molecular size and rotatable bond distributions; DFT calculation times; prediction error metrics; solvent and spin–orbit coupling effects on ^31^P NMR shifts (PDF)

## References

[1] Keith J. A., Vassilev-Galindo V., Cheng B., Chmiela S., Gastegger M., Müller K.-R., Tkatchenko A. (2021). Combining Machine Learning and Computational
Chemistry for Predictive Insights Into Chemical Systems. Chem. Rev..

[2] Volk A. A., Epps R. W., Yonemoto D. T., Masters B. S., Castellano F. N., Reyes K. G., Abolhasani M. (2023). AlphaFlow:
autonomous discovery and
optimization of multi-step chemistry using a self-driven fluidic lab
guided by reinforcement learning. Nat. Commun..

[3] Mirza A., Alampara N., Kunchapu S., Ríos-García M., Emoekabu B., Krishnan A., Gupta T., Schilling-Wilhelmi M., Okereke M., Aneesh A., Asgari M., Eberhardt J., Elahi A. M., Elbeheiry H. M., Gil M. V., Glaubitz C., Greiner M., Holick C. T., Hoffmann T., Ibrahim A., Klepsch L. C., Köster Y., Kreth F. A., Meyer J., Miret S., Peschel J. M., Ringleb M., Roesner N. C., Schreiber J., Schubert U. S., Stafast L. M., Wonanke A. D. D., Pieler M., Schwaller P., Jablonka K. M. (2025). A framework for
evaluating the chemical knowledge and reasoning abilities of large
language models against the expertise of chemists. Nat. Chem..

[4] Wu T., Kheiri S., Hickman R. J., Tao H., Wu T. C., Yang Z.-B., Ge X., Zhang W., Abolhasani M., Liu K., Aspuru-Guzik A., Kumacheva E. (2025). Self-driving lab for the photochemical
synthesis of plasmonic nanoparticles with targeted structural and
optical properties. Nat. Commun..

[5] Steinbeck C. (2001). SENECA: A
platform-independent, distributed, and parallel system for computer-assisted
structure elucidation in organic chemistry. J. Chem. Inf. Comput. Sci..

[6] Elyashberg M., Argyropoulos D. (2021). Computer Assisted
Structure Elucidation (CASE): Current
and future perspectives. Magn. Reson. Chem..

[7] Nuzillard J.-M., Georges M. (1991). Logic for structure
determination. Tetrahedron.

[8] Jonas E., Kuhn S., Schlörer N. (2022). Prediction
of chemical shift in NMR:
A review. Magn. Reson. Chem..

[9] Kuhn S., Kolshorn H., Steinbeck C., Schlörer N. (2024). Twenty years
of nmrshiftdb2: A case study of an open database for analytical chemistry. Magn. Reson. Chem..

[10] Hack J., Jordan M., Schmitt A., Raru M., Zorn H. S., Seyfarth A., Eulenberger I., Geitner R. (2023). Ilm-NMR-P31: an open-access
31P nuclear magnetic resonance database and data-driven prediction
of 31P NMR shifts. J. Cheminform..

[11] Ramsey N. F. (1950). Magnetic
Shielding of Nuclei in Molecules. Phys. Rev..

[12] Ramsey N. F. (1953). Electron
Coupled Interactions between Nuclear Spins in Molecules. Phys. Rev..

[13] Gao P., Zhang J., Chen H. (2021). A systematic benchmarking of 31 P
and 19 F NMR chemical shift predictions using different DFT/GIAO methods
and applying linear regression to improve the prediction accuracy. Int. J. Quantum Chem..

[14] Payard P.-A., Perego L. A., Grimaud L., Ciofini I. (2020). A DFT Protocol for
the Prediction of 31 P NMR Chemical Shifts of Phosphine Ligands in
First-Row Transition-Metal Complexes. Organometallics.

[15] Kondrashova S. A., Polyancev F. M., Latypov S. K. (2022). DFT Calculations of 31P NMR Chemical
Shifts in Palladium Complexes. Molecules.

[16] Grimme S., Bannwarth C., Dohm S., Hansen A., Pisarek J., Pracht P., Seibert J., Neese F. (2017). Fully Automated Quantum-Chemistry-Based
Computation of Spin-Spin-Coupled Nuclear Magnetic Resonance Spectra. Angew. Chem., Int. Ed..

[17] Shenderovich I. G. (2021). Experimentally
Established Benchmark Calculations of 31 P NMR Quantities. Chemistry-Methods.

[18] van
Wüllen C. (2000). A comparison of density functional methods for the
calculation of phosphorus-31 NMR chemical shifts. Phys. Chem. Chem. Phys..

[19] Maryasin B., Zipse H. (2011). Theoretical studies
of 31P NMR spectral properties of phosphanes
and related compounds in solution. Phys. Chem.
Chem. Phys..

[20] Schattenberg C. J., Kaupp M. (2021). Extended Benchmark Set of Main-Group Nuclear Shielding Constants
and NMR Chemical Shifts and Its Use to Evaluate Modern DFT Methods. J. Chem. Theory Comput..

[21] Calcagno F., Maryasin B., Garavelli M., Avagliano D., Rivalta I. (2024). Modeling solvent effects and convergence
of 31P-NMR
shielding calculations with COBRAMM. J. Comput.
Chem..

[22] Streck R., Barnes A. J. (1999). Solvent effects on infrared, 13C and 31P NMR spectra
of trimethyl phosphate. Spectrochim. Acta, A
Mol. Biomol. Spectrosc..

[23] Gensch T., Dos Passos Gomes G., Friederich P., Peters E., Gaudin T., Pollice R., Jorner K., Nigam A., Lindner-D’Addario M., Sigman M. S., Aspuru-Guzik A. (2022). A Comprehensive Discovery Platform
for Organophosphorus Ligands for Catalysis. J. Am. Chem. Soc..

[24] Paruzzo F. M., Hofstetter A., Musil F., De S., Ceriotti M., Emsley L. (2018). Chemical shifts
in molecular solids by machine learning. Nat.
Commun..

[25] Cordova M., Engel E. A., Stefaniuk A., Paruzzo F., Hofstetter A., Ceriotti M., Emsley L. (2022). A Machine
Learning Model of Chemical
Shifts for Chemically and Structurally Diverse Molecular Solids. J. Phys. Chem. C.

[26] RDKit: Open-Source Cheminformatics; RDKit, 2025.

[27] Ermanis K., Parkes K. E. B., Agback T., Goodman J. M. (2019). The optimal DFT
approach in DP4 NMR structure analysis - pushing the limits of relative
configuration elucidation. Org. Biomol. Chem..

[28] Weininger D. (1988). SMILES, a
chemical language and information system. 1. Introduction to methodology
and encoding rules. J. Chem. Inf. Comput. Sci..

[29] Landrum, G. ; Tosco, P. ; Kelley, B. ; Ric; Cosgrove, D. ; Sriniker; Gedeck; Vianello, R. ; Schneider, N. ; Kawashima, E. ; Dan, N. ; Gareth, J. ; Dalke, A. ; Cole, B. ; Swain, M. ; Turk, S. ; Savelyev, A. ; Vaucher, A. ; Wójcikowski, M. ; Take, I. ; Probst, D. ; Ujihara, K. ; Scalfani, V. F. ; Godin, G. ; Lehtivarjo, J. ; Walker, R. ; Pahl, A. ; Berenger, F. ; Jasondbiggs; strets123 rdkit/rdkit: _03_3 (Q1 ) Release; Zenodo 2023. https://www.rdkit.org/2023.

[30] Riniker S., Landrum G. A. (2015). Better Informed
Distance Geometry: Using What We Know
To Improve Conformation Generation. J. Chem.
Inf. Model.

[31] Wang S., Witek J., Landrum G. A., Riniker S. (2020). Improving
Conformer
Generation for Small Rings and Macrocycles Based on Distance Geometry
and Experimental Torsional-Angle Preferences. J. Chem. Inf. Model..

[32] Neese F. (2022). Software update:
The ORCA program system—Version 5.0. WIREs Comput. Mol. Sci..

[33] Neese F. (2003). An improvement
of the resolution of the identity approximation for the formation
of the Coulomb matrix. J. Comput. Chem..

[34] Neese F. (2023). The SHARK
integral generation and digestion system. J.
Comput. Chem..

[35] Becke A. D. (1988). Density-functional
exchange-energy approximation with correct asymptotic behavior. Phys. Rev. A.

[36] Lee C., Yang W., Parr R. G. (1988). Development
of the Colle-Salvetti
correlation-energy formula into a functional of the electron density. Phys. Rev. B.

[37] Weigend F., Ahlrichs R. (2005). Balanced basis sets of split valence, triple zeta valence
and quadruple zeta valence quality for H to Rn: Design and assessment
of accuracy. Phys. Chem. Chem. Phys..

[38] Caldeweyher E., Bannwarth C., Grimme S. (2017). Extension of the D3 dispersion coefficient
model. J. Chem. Phys..

[39] Stephens P. J., Devlin F. J., Chabalowski C. F., Frisch M. J. (1994). Ab Initio Calculation
of Vibrational Absorption and Circular Dichroism Spectra Using Density
Functional Force Fields. J. Phys. Chem. A.

[40] Becke A. D. (1993). Density-functional
thermochemistry. III. The role of exact exchange. J. Chem. Phys..

[41] Wolinski K., Hinton J. F., Pulay P. (1990). Efficient
implementation of the gauge-independent
atomic orbital method for NMR chemical shift calculations. J. Am. Chem. Soc..

[42] Ditchfield R. (1974). Self-consistent
perturbation theory of diamagnetism. Mol. Phys..

[43] Keal T. W., Tozer D. J. (2004). A semiempirical generalized gradient approximation
exchange-correlation functional. J. Chem. Phys..

[44] Furness J.
W., Kaplan A. D., Ning J., Perdew J. P., Sun J. (2020). Accurate and
Numerically Efficient r2SCAN Meta-Generalized Gradient Approximation. J. Phys. Chem. Lett..

[45] van
Lenthe E., Snijders J. G., Baerends E. J. (1996). The zero-order regular
approximation for relativistic effects: The effect of spin–orbit
coupling in closed shell molecules. J. Chem.
Phys..

[46] Pantazis D. A., Chen X.-Y., Landis C. R., Neese F. (2008). All-Electron Scalar
Relativistic Basis Sets for Third-Row Transition Metal Atoms. J. Chem. Theory Comput..

[47] Pracht P., Grimme S., Bannwarth C., Bohle F., Ehlert S., Feldmann G., Gorges J., Müller M., Neudecker T., Plett C., Spicher S., Steinbach P., Wesołowski P. A., Zeller F. (2024). CREST-A program for
the exploration
of low-energy molecular chemical space. J. Chem.
Phys..

[48] Bannwarth C., Ehlert S., Grimme S. (2019). GFN2-xTB-An
Accurate and Broadly
Parametrized Self-Consistent Tight-Binding Quantum Chemical Method
with Multipole Electrostatics and Density-Dependent Dispersion Contributions. J. Chem. Theory Comput..

[49] Howarth A., Goodman J. M. (2022). The DP5 probability,
quantification and visualisation
of structural uncertainty in single molecules. Chem. Sci..

[50] Kosolapoff, G. M. ; Maier, L. Organic Phosphorus Compounds, 2nd ed.; Wiley, 1973.

[51] R Core team . R: A Language and Environment for Statistical Computing; R Foundation for Statistical Computing, 2022. http://www.r-project.org/.

[52] Cao Y., Charisi A., Cheng L.-C., Jiang T., Girke T. (2008). ChemmineR:
a compound mining framework for R. Bioinformatics.

[53] Horan, Kevin. ; Girke, Thomas. ChemmineOB: R Interface To A Subset of OpenBabel Functionalities; github, 2022. https://github.com/girke-lab/ChemmineOB.

[54] O’Boyle N. M., Banck M., James C. A., Morley C., Vandermeersch T., Hutchison G. R. (2011). Open Babel:
An open chemical toolbox. J. Cheminform..

[55] Ushey, K. ; Allaire, J. J. ; Tang, Y. reticulate: Interface to ’Python’; CRAN, 2023. https://cran.r-project.org/package=reticulate.

[56] Pupier M., Nuzillard J.-M., Wist J., Schlörer N. E., Kuhn S., Erdelyi M., Steinbeck C., Williams A. J., Butts C., Claridge T. D. W., Mikhova B., Robien W., Dashti H., Eghbalnia H. R., Farès C., Adam C., Kessler P., Moriaud F., Elyashberg M., Argyropoulos D., Pérez M., Giraudeau P., Gil R. R., Trevorrow P., Jeannerat D. (2018). NMReDATA, a standard to report the NMR assignment and
parameters of organic compounds. Org. Magn.
Reson..

[57] Müller, K. ; Wickham, H. tibble: Simple Data Frames; CRAN, 2022. https://cran.r-project.org/package=tibble.

