# Supplementary material for: Predicting 31P NMR Shifts in Large-Scale, Heterogeneous Databases by Gas Phase DFT: Impact of Conformer and Solvent Effects
Source: ACS Omega. 2026 Jan 16;11(4):6773–82. doi: 10.1021/acsomega.5c13249 (PMC12878327; doi:10.1021/acsomega.5c13249)
Supplement: Supplementary file 1 [file ao5c13249_si_001.pdf]

Predicting ^{31}P NMR Shifts in Large-Scale, Heterogeneous Databases by Gas Phase DFT: Impact of Conformer and Solvent Effects

Robert Geitner[a,*], Christian Dreßler[b]

[a] Group for Physical Chemistry/Catalysis, Department of Mathematics and Natural Sciences, Institute of Chemistry and Bioengineering, Technische Universität Ilmenau, Weimarer Str. 32, 98693 Ilmenau, Germany, *e-mail: robert.geitner@tu-ilmenau.de

[b] Group for Solid State Physics, Department of Mathematics and Natural Sciences, Institute of Physics, Technische Universität Ilmenau, Weimarer Str. 32, 98693 Ilmenau, Germany

1. Dataset Description

The number of molecules for which quantum chemical derived ^{31}P NMR shifts are reported is 10,007, of which 9,963 are organic in nature and only 44 are inorganic, meaning that they do not contain a carbon atom. The average molecule consists of 36 atoms and has 4 rotatable bonds. The distribution of both parameters is also shown in **Figure S1**.

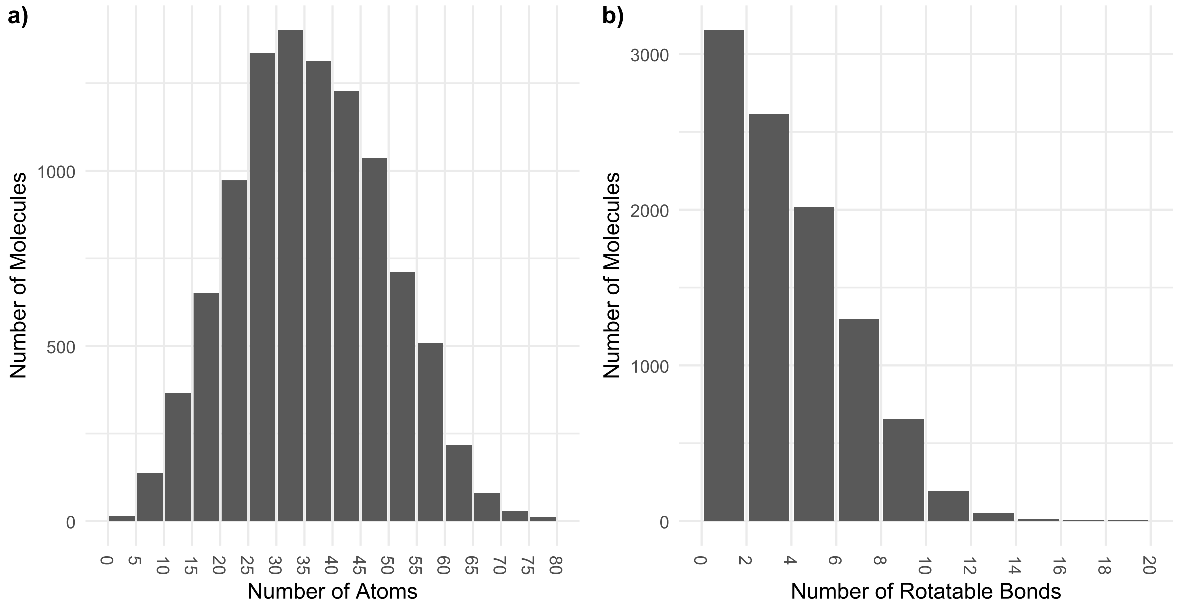

Figure S1. *Distribution of the number of atoms (a) and of number of rotatable bonds (b) in the dataset.*

Table S1. *Calculation time t_C for geometry optimization and NMR single point calculation using 16 CPUs (Intel Xeon Platinum 8462Y, 2.8 GHz) using C-PCM implicit solvent models (chloroform, dimethyl sulfoxide, toluene, water, and acetonitrile).*

**Quan-tile**	**Size**	**Atom Range**	**t_C(vac) / min**	**t_C($CHCl_3$) / min**	**t_C(DMSO) / min**	**t_C(Tol.) / min**	**t_C(H_2O) / min**	**t_C(ACN) / min**
**Q1**	2021	3-25	5.2	5.5	5.9	5	6.2	6.1
**Q2**	2021	25-32	9.8	10.9	11.6	10	12	11.7
**Q3**	2021	32-40	15.1	17.9	18.6	16.5	19.6	19.2
**Q4**	2021	40-48	23.3	28.7	29.4	26.7	30.9	30.3
**Q5**	2020	48-80	39.7	50.2	51.1	47.5	53.2	53.3

Table S2. *Correlation between the number of rotatable bonds and DFT-derived prediction accuracy of ^{31}P NMR shifts.*

**Quantile**	**Size**	**Rot. Bond Range**	**$RMSE_{bol}$ / ppm**	**$RMSE_{vac}$ / ppm**	**μ(# of Conf.)**	**μ(# of Atoms)**
**Q1**	2002	0-2	35.9	36.7	10	24.8
**Q2**	2002	2-3	33.1	34.4	30	32.3
**Q3**	2001	3-5	25.1	27.1	77	35.9
**Q4**	2001	5-7	25.0	28.5	234	40.8
**Q5**	2001	7-22	26.0	26.0	1424	48.7

Table S3. *Influence of implicit solvent models on the DFT-derived prediction accuracy of ^{31}P NMR shifts. Extension to Table 1 by providing standard deviations for the MAE as well as the MSE.*

Exp. Solvent	# of Mol.	Impl. Solvent	MAE / ppm	σ(MAE) / ppm	MSE / ppm
**-**	10,007	-	18.22	24.85	-3.30
		Boltzm.-weight.	16.80	24.08	-3.42
**$CHCl_3$**	2,955	-	15.42	22.15	+3.63
		$CHCl_3$	13.90	22.62	+0.64
**DMSO**	43	-	20.04	17.19	+6.81
		DMSO	12.81	21.90	-3.53
**Toluene**	11	-	23.94	8.68	-4.74
		Toluene	21.75	7.94	-5.16
**H_2O**	205	-	15.09	26.41	-2.53
		H_2O	14.08	24.87	-7.91
**CH_3CN**	4	-	25.89	35.00	-16.52
		CH_3CN	25.53	36.74	-19.33

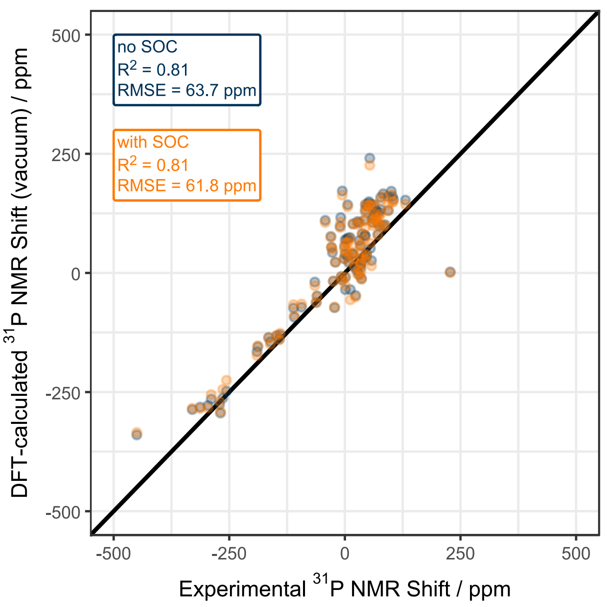

Figure S2. *Parity plot of DFT-calculated ^{31}P NMR shifts vs experimental ^{31}P shifts. The shifts were calculated with and without spin orbit coupling (SOC) for 105 molecules containing Sn, Te and I besides lighter elements.*
